# The Improved Microwave Absorption Performance of the 3D Porous (Ni@NO-C)_n_/NO-C Composite Absorber

**DOI:** 10.3390/nano13202772

**Published:** 2023-10-16

**Authors:** Xinmeng Jia, Zhigang Li, Chao Ruan, Yongfu Lian

**Affiliations:** 1 Key Laboratory of Functional Inorganic Material Chemistry, Ministry of Education, School of Chemistry and Materials Science, Heilongjiang University, Harbin 150080, China2221367@s.hlju.edu.cn (C.R.); 2 Heilongjiang Institute of Atomic Energy, Harbin 150086, China; lzg0015@hrbeu.edu.cn

**Keywords:** NO-doped carbon, magnetic metal, special microstructure, impedance match, reflection loss

## Abstract

Microwave absorbers that are lightweight and have good stability and high efficiency have attracted much attention for their applications in many contemporary fields. In this work, a 3D porous (Ni@NO-C)_n_/NO-C composite absorber was prepared using a wet chemistry method with Ni chains and melamine as precursors, in which NO-C (N,O-doped carbon)-encapsulated Ni particles are homogenously dispersed in the 3D porous networks of NO-C in the form of (Ni@NO-C)_n_ chains. The special microstructure of the as-prepared material is proven to be beneficial for the improvement of its microwave absorption performance. The as-synthesized (Ni@NO-C)_n_/NO-C composite absorber exhibited an effective absorption bandwidth of 4.1 GHz and an extremely large reflection loss of −72.3 dB. The excellent microwave-absorbing performances can be ascribed to the cooperative consequences of dielectric loss and magnetic loss, along with the balance between attenuation capability and impedance matching.

## 1. Introduction

Microwave absorbers (MAs) have attracted much attention for their applications in military defense, information safety, wireless data communication, etc. [1]. In contrast to traditional electromagnetic shielding reflection materials, MAs can convert electromagnetic waves into heat and other kinds of energy, or dissipate them through interference [2]. In line with classical electromagnetic theory, electromagnetic waves are composed of a rapidly oscillating electric field and a magnetic field [3], and the absorption characteristics of MAs are highly dependent on their dielectric loss, magnetic loss, and impedance matching [4]. Normally, the MAs with the largest dielectric losses are barium titanate ceramics [5], silicon carbide [6], conductive polymers [7], carbon materials [8], and transition metal sulfides [9], whereas those with the greatest magnetic losses are mainly composed of magnetic metals and their alloys or compounds [10,11]. 

To date, MAs with a single dielectric or magnetic loss are far from meeting the rapidly expanding demands, and an effective way of improving the performance of electromagnetic wave absorption is the construction of dual-/multiple-component composite MAs, making full use of the loss mechanisms of various materials [12]. As one of the most typical dual-component composite MAs, magnetic metal/carbon composites have become a research focus in the field of electromagnetic wave absorption. Carbon materials, a kind of very important dielectric loss MA, have the advantages of low density, excellent chemical stability, diverse microscopic morphology, adjustable dielectric constant, strong dielectric loss ability, etc. [13]. On the other hand, magnetic metals usually show a strong magnetic loss ability because of their high saturation magnetization, Snoek limit, and low coercivity [14]. It is expected that the composite of magnetic metals and carbon materials can not only maintain the magnetic losses of magnetic metals, but also strengthen the dielectric losses of carbon materials via high-intensity interface polarization, dipolar-oriented polarization, and other polarization relaxation mechanisms. Moreover, the impedance-matching characteristics of the magnetic metal/carbon composites could be optimized by the regulation of their microstructures and component metal/carbon ratios [15,16], and carbon materials are composited with magnetic metals in the form of coating, which can not only slow down the oxidation and corrosion rate of metal materials but also reduce the overall density of composite materials. Therefore, researchers have conducted a lot of research on the preparation of magnetic metal/carbon composites. Li et al. [17] fabricated yolk−shell-structured Co_3_Fe_7_@C with the precursor of (Co_0.9_Fe_0.1_) Fe_2_O_4_@phenolic resin, in which the carbon shell can effectively prevent the oxidation and agglomeration of encapsulated metal alloy particles. Shen et al. [18] prepared porous Ni/NiO/carbon nanofibers with successive electrospinning, vacuum calcination, and chemical etching, which exhibited a maximum reflection loss of −47.9 dB at 9.5 GHz. Sha et al. [19] constructed chemical Ni−C bonding using a microwave welding method at the interface between carbon nanotubes and Ni nanoparticles, which could induce a strong microwave absorption band in the range of 10 to 18 GHz. Ding et al. [20] synthesized Co-nanoparticle-loaded carbon nanosheets via a simple swelling and carbonization treatment of cellulose fibrils, and confirmed that the anisotropy derived from the lamellar structure of two-dimensional carbon materials was responsible for the magnetic resonance. Qi et al. [21] prepared a composite of Co/CoO/LSC (lotus seedpod carbon) with the carbonization of lotus seedpods and hydrogen reduction of Co_3_O_4_, in which the 3D porous biomass-derived carbon was proven to be conducive to microwave absorption for the improvement of impedance match and multiple scattering. All of the above works are based on the composites of 0D magnetic metals (or their alloys and oxides) and 0–3D carbon materials. Recently, Zhao et al. [22] fabricated a triple-component composite of graphene aerogels, carbon nanotubes, and CoNi chains (GA-CNT-CoNi) through a facile freeze-drying strategy followed by thermal reduction, which demonstrated a superior EM wave absorption performance because of the bridging roles played by 1D CoNi chains and CNTs to link graphene nanosheets for the construction of a conductive and macroscopic network. 

On the basis of structural engineering, the heteroatom doping/modification of carbon materials offers an effective approach to adjusting the microwave absorption properties of the composites of magnetic metals/carbon materials [23,24]. Different from carbon atoms in terms of atomic size, bond length, number of nuclear electrons, and electron spin density, heteroatoms, e.g., B, N, P, and S, could introduce a large number of point defect groups in the vicinity of carbon atoms, changing the surface electronic structure and forming abundant defect dipoles to enhance dipole polarization in carbon materials [25]. Wang et al. [26] synthesized novel bowknot-like N-doped and carbon-encapsulated Co nanoparticles, which exhibited an excellent absorbing performance with a minimum reflection loss of −47.6 dB at 11.0 GHz. Zhang et al. [27] fabricated hollow N-doped carbon polyhedron (NCP)-encapsulated CoNi@NC nanoparticles with zeolitic imidazolate frameworks-67 (ZIF-67) as a solid template, and speculated that the C−N species on the surfaces of N-doped carbon contributed a lot to the dipole polarizations. Liu et al. [28] prepared cobalt nanoparticles decorated with N-doped carbon nanofibers (Co/N-C NFs) using electrospinning and annealing methods, whose excellent microwave absorption properties were ascribed to proper impedance matching via adjusting the composition of the absorber and a higher Co content. In addition, boron and nitrogen co-doped graphene nanosheets (BCN) [29] as well as sulfur-doped graphene (S-GS) [30] were also prepared, in which the contributions of heteroatom doping to the microwave absorption properties of graphene were verified.

Herein, Ni chains were initially synthesized using a wet chemical method with the assistance of a hydrophilic polymer template. Subsequently, Ni chains and melamine were annealed in an inert atmosphere, and a composite microwave absorber consisting of NO-C-encapsulated Ni nanoparticles (Ni@NO-C) and 3D NO-C was obtained. The as-prepared Ni/NO-C composite has a special microstructure and displays a largely improved microwave absorption performance both in the maximum reflection loss and in the effective absorption bandwidth, which might largely owe to the in situ-grown (Ni@NO-C)_n_ chains and the 3D porous networks of NO-C. The novel porous 3D magnetic metal/carbon composite fabricated in this work has advantages over the previously reported 3D porous Ni chain networks [31] in terms of lighter weight, better stability and more effective microwave absorption efficiency.

## 2. Materials and Methods

### 2.1. Materials

Nickel chloride hexahydrate (NiCl_2_·6H_2_O), hydrazine monohydrate (N_2_H_4_·H_2_O), and polyvinylpyrrolidone (PVP) were purchased from Shanghai Macklin Biochemical Co., Ltd. (Shanghai, China). Melamine, ethylene glycol (EG), anhydrous ethanol, and ethanol were purchased from Tianjin Fuyu Fine Chemical Co., Ltd. (Tianjin, China). All the chemicals were analytical reagents and used without further purification. 

### 2.2. Preparation of Ni Nanoparticle-Assembled Chains

Ni nanoparticle-assembled chains were prepared using a templated wet chemical method [31] with PVP as the soft template and ethylene glycol as the solvent. Typically, after 1.08 g NiCl_2_·6H_2_O and 4 g PVP were completely dissolved in 300 mL ethylene glycol a mixture of 1 mL hydrazine hydrate and 9 mL anhydrous ethanol was added dropwise under intensive stirring. With the color of the solution changed from grass green to blue, the reaction mixture was kept stirring for 30 min and then transferred to a three-necked flask. After refluxing for 1 h gray participates occurred and then were magnetically separated and successively washed with deionized water and ethanol several times. Ni nanoparticle-assembled chains were obtained after the participates were dried at 313 K in a vacuum oven for 12 h. 

### 2.3. Fabrication of 3D Porous (Ni@NO-C)_n_/NO-C Composite Absorber

In total, 0.2 g Ni nanoparticle-assembled chains and 2.5 g melamine were homogeneously mixed in an agate mortar by vigorous grinding for 15 min, and then transferred to a quartz boat. Under the protection of argon gas, the powder mixture was heated in a tubular furnace to 750 °C at a heating rate of 5 °C/min and then kept stable for 3 h. When the obtained black mixture was naturally cooled down, a 3D porous (Ni@NO-C)_n_/NO-C composite absorber was collected.

### 2.4. Characterization Methods

SEM and TEM observations were conducted on a field-emission scanning electron microscope (QUANTA 200S, FEI, Eindhoven, Holland) and a transmission electron microscope (JEM2100, JEOL, Yokyo, Japan), respectively. Data of X-ray diffraction were collected using an X-ray diffractometer (D8 Advance, Bruker, Berlin, Germany), and the Raman scattering spectrum was recorded on a confocal Raman spectroscopic system (HR 800, Jobin Yvon, Palaiseau, France) excited with a laser of 458 nm. The elemental composition and functional groups of materials were analyzed by an X-ray photoelectron spectrometer (Kratos- Axis Ultra DLD, Shimadzu, Kyoto, Japan).

### 2.5. Electromagnetic Parameter Test

The electromagnetic parameters of the 3D porous (Ni@NO-C)_n_/NO-C composite absorber were obtained by a microwave vector network analyzer (N5230A, Agilent, Santa Clara, CA, USA). At the temperature of 70 °C, the (Ni@NO-C)_n_/NO-C composite absorber was dispersed in the base material (paraffin wax) with a mass ratio of 30:70 (composite absorber:paraffin wax), and then molded into a ring sample with an inner diameter of 3.00 mm, an outer diameter of 7.00 mm and a thickness of 2 mm. Electromagnetic parameters were collected in the frequency band of 2–18 GHz using the coaxial line method, and the permittivity and permeability were calculated using the Nicolson–Ross–Weir (NRW) algorithm.

## 3. Results

**Figure 1:**
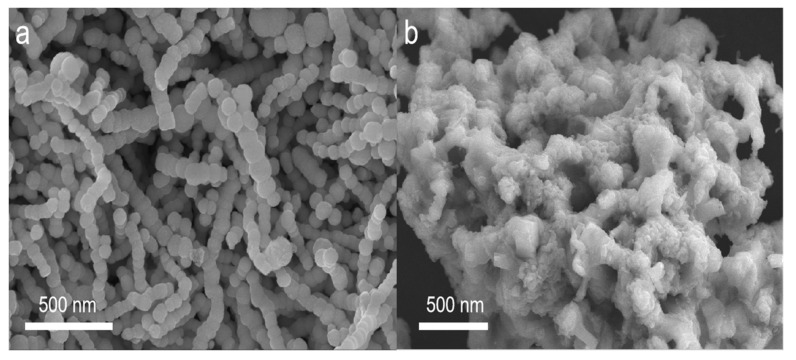
SEM images of the as-prepared Ni chains (**a**) and 3D porous (Ni@NO-C)_n_/NO-C composite absorber (**b**).

The microstructure of the as-prepared Ni chains and 3D porous (Ni@NO-C)_n_/NO-C composite absorber was characterized through SEM observations. As shown in Figure 1a, spherical Ni nanoparticles whose mean diameter was about 100 nm assembled to form bead chains with various lengths under the assistance of PVP molecular templates. In comparison with those reported previously [31], smaller Ni particles and shorter bead chains were detected. Instead of aggregating to 3D nickel chain nets through cross-linking with each other to form longer nickel chains, the shorter Ni chains showed smoother surfaces and were well isolated. It can be seen from Figure 1b that after pyrolysis at 750 °C with melamine, the Ni chains were coated with NO-C materials derived from melamine. Moreover, the NO-C-coated Ni chains and NO-doped carbon materials form 3D porous networks, in which Ni chains were homogeneously dispersed in the porous nets of NO-C materials. On one hand, these magnetic metal/carbon composite materials with a novel microstructure could act as a kind of microwave absorbers with high stability and light weight. On the other hand, they could also be a kind of microwave absorbers with high electromagnetic wave absorption efficiency, because their adjustable conductivity would lead to a balance between impedance matching and attenuation capability.

**Figure 2:**
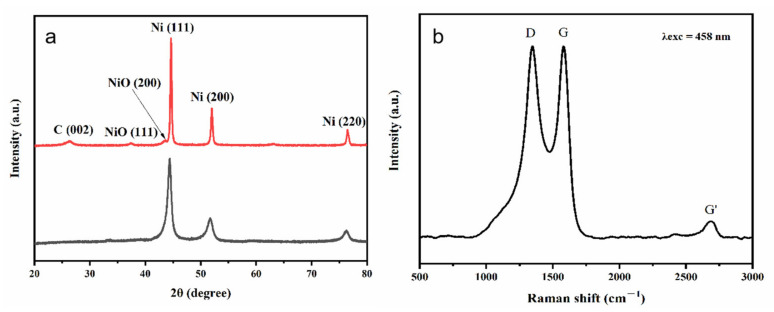
The XRD patterns of Ni chains (black) and the 3D porous (Ni@NO-C)_n_/NO-C composite absorber (red) (**a**) and Raman spectrum of the 3D porous (Ni@NO-C)_n_/NO-C composite absorber (**b**).

Shown in Figure 2a are the powder X-ray diffraction (XRD) patterns of Ni chains and the 3D porous (Ni@NO-C)_n_/NO-C composite absorber, in which the strong diffraction peaks observed at 44.5°, 51.9°, and 76.4°could be indexed to the (111), (200), and (220) lattice planes of fcc–Ni (JCPDS 04-0850), respectively. For Ni chains, no other diffraction peak was observed, indicating that the prepared Ni chains are of single phase and not obviously oxidized. For the 3D porous (Ni@NO-C)_n_/NO-C composite absorber, the weak diffraction peaks detected at 37.5°, 43.4°, and 63.2° could be assigned to the (111), (200), and (220) lattice planes of fcc–NiO (JCPDS 47-1049), respectively. The small diffraction peak detected at 26.5° is ascribed to the (002) lattice plane of graphite, confirming that the carbon component in melamine was partially graphitized. In line with the Mering–Maire equation [32], the degree of graphitization was calculated to be 46.1%. Additionally, the full width at half maximum (FWHM) of the 3D porous (Ni@NO-C)_n_/NO-C composite absorber is much narrower than that of Ni chains, implying that the crystallite size of Ni particles in the former could be expected to be much larger than that in the latter.

In the Raman spectrum of the 3D porous (Ni@NO-C)_n_/NO-C composite absorber displayed in Figure 2b, the featured D, G, and G’ bands of carbon materials are clearly observed at 1350, 1582, and 2700 cm^−1^, respectively. Based on its position (around 2700 cm^−1^) and the multiple secondary structures of G’ band, it can be concluded that multilayered graphite is formed via the pyrolysis of melamine. Particularly, the disorder-induced D band is normally associated with the presence of in-plane substitutional heteroatoms, vacancies, grain boundaries, or other defects, and the intensity ratio of the D and G bands (*I_D_/I_G_*) has been regarded as a direct indicator of the crystalline symmetry of carbon materials [33]. The *I_D_/I_G_* in the area of integral intensity is measured to be 1.6, indicative of the plentiful defects and low crystalline symmetry of the carbon materials in the 3D porous (Ni@NO-C)_n_/NO-C composite absorber.

**Figure 3:**
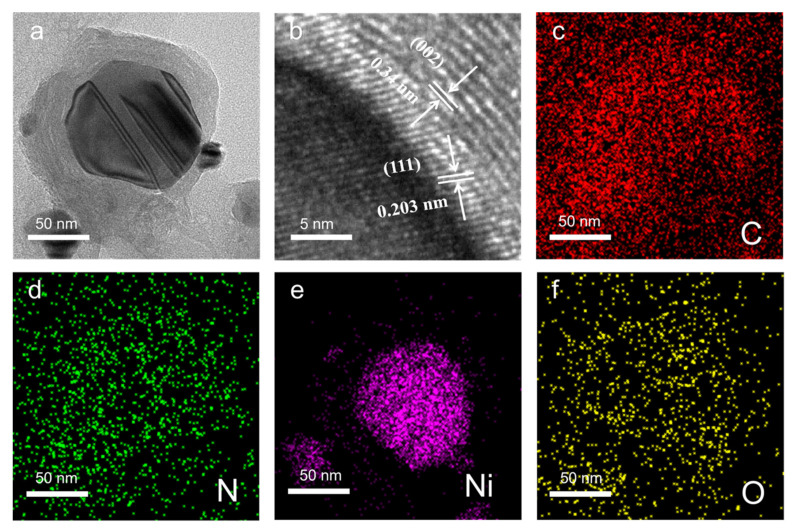
TEM (**a**), HR-TEM (**b**), and elemental mapping (**c**–**f**) images of 3D porous (Ni@NO-C)_n_/NO-C composite absorber.

Displayed in Figure 3a is a typical TEM image of a Ni@NO-C particle in the 3D porous (Ni@NO-C)_n_/NO-C composite absorber. It is obvious that a Ni nanoparticle is encapsulated in the NO-C materials, and that the crystalline nickel core and partially graphited NO-C layers could be clearly distinguished. This core–shell-structured Ni@NO-C composite particles endowed the composite of Ni chains and NO-C with much improved stability. From the HR-TEM image displayed in Figure 3b, it can be observed that the lattice spacing of the crystalline Ni core is 0.203 nm, corresponding to the (111) lattice plane of the fcc–Ni crystal, whereas the lattice spacing of the partially graphited NO-C layers is around 0.34 nm, corresponding to the (002) lattice plane of graphite. It should be noted that incomplete carbon layers grow unevenly around the Ni nanoparticle, which might be a result of a competitive effect among the neighboring Ni particles in a Ni chain. Moreover, the elemental mapping images shown in Figure 3c–f evidence that the core is made of nickel and that nitrogen and oxygen are evenly distributed in the partially graphited carbon layers, offering solid support both for the elemental composition and for the core–shell structure of the Ni@NO-C composite particle.

**Figure 4:**
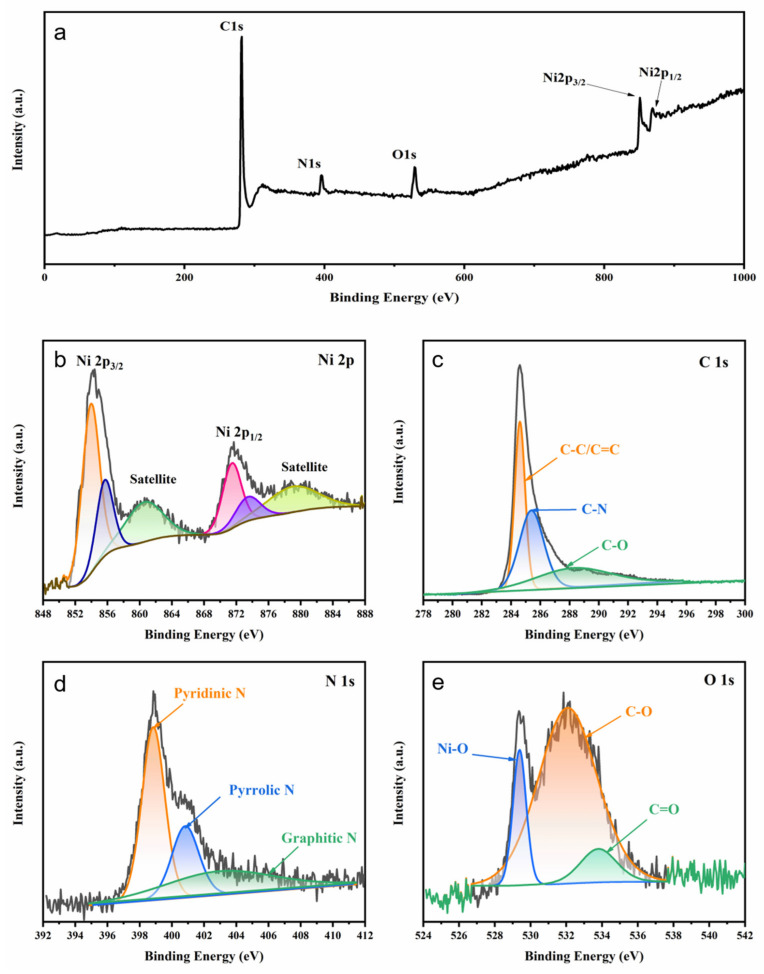
The survey (**a**) and deconvoluted Ni 2p (**b**), C 1s (**c**), N 1s (**d**), and O 1s (**e**) XPS spectra of the 3D porous (Ni@NO-C)_n_/NO-C composite absorber.

The surface chemical state and elemental composition of the 3D porous (Ni@NO-C)_n_/NO-C composite absorber were investigated by XPS. From the survey XPS spectrum illustrated in Figure 4a, it can be seen that elements including Ni, C, N and O were present in the composite of Ni chains and N,O-doped carbon materials, whose relative molar contents have been estimated to be 17.25%, 69.96%, 8.70%, and 4.09%, respectively. In the deconvoluted Ni 2p spectrum (Figure 4b), the two main peaks at 853.9 and 871.6 eV correspond to the characteristic bonding energies of Ni 2p_3/2_ and Ni 2p_1/2_ for Ni, whereas the two peaks at 855.9 and 873.7 eV correspond to the characteristic bonding energies of Ni 2p_3/2_ and Ni 2p_1/2_ for NiO, and the two satellites detected around 860.8 and 879.5 eV offer further support for the presence of Ni atoms and Ni^2+^ ions in the 3D porous (Ni@NO-C)_n_/NO-C composite absorber. In the deconvoluted C 1s spectrum (Figure 4c), the peaks at 284.6, 285.4, and 288.5 eV are ascribed to C-C/C=C, C-N, and C-O, respectively, indicating that the partially graphitized carbon layers are co-doped with nitrogen and oxygen elements. In the deconvoluted N 1s spectrum (Figure 4d), the peaks at 398.8, 400.8, and 403.1 eV are related to the pyridinic, pyrrolic, and graphitic nitrogen atoms, respectively. It is important to note that the majority of nitrogen atoms are doped in the margins (pyridinic N) or defects (pyrrolic N) of the partially graphitized carbon layers. In the deconvoluted O 1s spectrum (Figure 4e), the peaks at 529.4, 532.1, and 533.8 eV can be attributed to Ni-O, C-O, and C = O, respectively. It is assumed that oxygen atoms are covalently bonded to the surface of partially graphited carbon layers, apart from those bonded with Ni. Thus, XPS characterization confirms the high-density margins led by nitrogen doping as well as oxygen-containing functional groups in the partially graphitized carbon layers.

**Figure 5:**
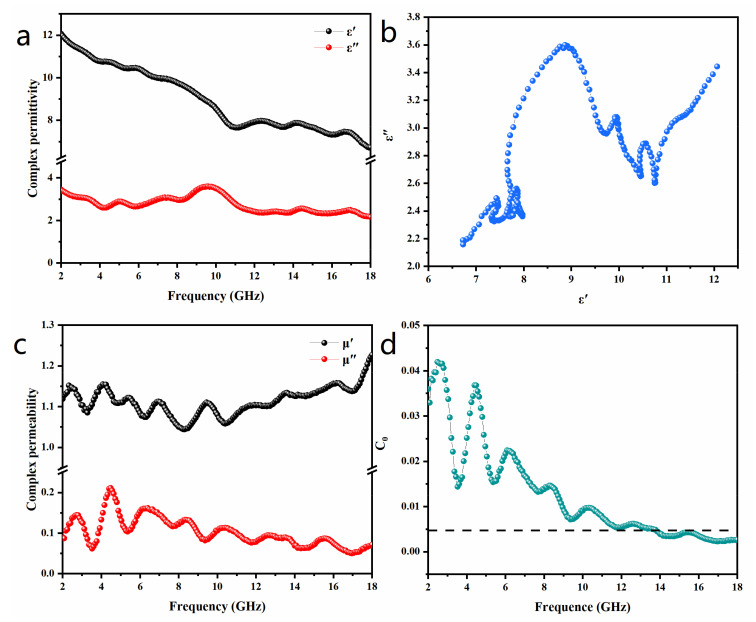
The complex permittivity (**a**), Cole–Cole curve (**b**), complex permeability (**c**), and *C*_0_ curve (**d**) of the paraffin-based composite with 30 wt. % (Ni@NO-C)_n_/NO-C.

When MAs were radiated by EM waves, the impedance matching between MAs and free space was crucial, since only those MAs that satisfy impedance matching will allow as much radiated EM wave as possible to enter their interiors, paving the way for the subsequent absorption of the EM wave. The complex permittivity and permeability of MAs play significant roles in microwave absorption. When the frequency of the EM wave and the thickness of the MAs are fixed, impedance matching is only related to the complex permittivity and the complex permeability of MAs. Figure 5a illustrates the frequency dependence of the complex permittivity for the paraffin-based composite with 30 wt. % (Ni@NO-C)_n_/NO-C over the 2.0–18.0 GHz range. The real part of complex permittivity depicts the electrical energy storage due to the polarization of the electrical dipole, and the imaginary part of permittivity stands for energy conversion caused by the relaxation of various polarizations. It can be inferred that the value of the real part (*ε*′) decreases largely from 12.06 at 2.0 GHz to 6.73 at 18.0 GHz with the increase in frequency, whereas the value of the imaginary part (*ε*″) changes from 3.44 to 2.16 in an oscillatory mode. In comparison with the real part (*ε*′), little variation is observed for the imaginary part (*ε*″) of the paraffin-based composite with 30 wt. % (Ni@NO-C)_n_/NO-C in the frequency range of 2–18 GHz, implying that the as-prepared EM absorbent has a relatively stable dielectric loss ability.

According to the Debye relaxation theory, the relationship between *ε*′ and *ε*″ could be described by the following equation [34,35,36]:

(1) $${\left(\varepsilon^{\prime }-\frac{\varepsilon_{s}+\varepsilon_{\infty }}{2}\right)}^{2}+{\left(\varepsilon^{\prime \prime }\right)}^{2}={\left(\frac{\varepsilon_{s}-\varepsilon_{\infty }}{2}\right)}^{2}$$

where *ε_s_* is the static dielectric constant and *ε_∞_* is the dielectric constant at an infinitely high frequency. Taking *ε*″ as the ordinate and *ε*′ as the abscissa, the obtained curve is called the Cole–Cole plot, in which each downward semicircle represents a dipole polarization relaxation process. In the Cole–Cole plot shown in Figure 5b, multiple semicircles are observed, confirming the existence of multiple dielectric relaxations in the paraffin-based composite with 30 wt. % (Ni@NO-C)_n_/NO-C. Moreover, a straight line is observed in the real part (ε′) range of 11.55– 12.06, implying that a conductive loss process also occurs. This conductive loss process corresponds to the real part (*ε*′) and the imaginary part (*ε*″) in the frequency range of 2.00–2.56 GHz (see Figure 5a) [37,38,39].

In line with free electron theory, the dielectric loss ability and relative complex permittivity of MAs are determined by their polarization relaxation and conductive loss behaviors. Polarization relaxation includes ionic polarization, electronic polarization, dipole orientation polarization and interfacial polarization. Since ionic polarization and electronic polarization usually occur in the high-frequency range of 103–106 GHz, the intrinsic electric dipole polarization and interfacial polarization play important roles in the polarization relaxation of MAs. The electrons undergoing dipolar polarization are usually associated with crystal defects, heterogeneous interfaces, and the regions where the polarization of the electron cloud occurs. For the (Ni@NO-C)_n_/NO-C absorbent, the dipoles originated from positive Ni^2+^ ions with their surrounding negative O^2−^ ions, N,O-containing functional groups, and plentiful defects in the partially graphited N,O-C layers contribute a lot to the real part (*ε*′) through dipolar polarization, and to the imaginary part (*ε*″) through dipole relaxation. On the other hand, interface polarization usually occurs at the interface of the heterogeneous medium [40]. The multiple hierarchical interfaces (the barriers at defects, edges/boundaries or seams) in the composite of paraffin and (Ni@NO-C)_n_/NO-C also contribute to the multiple resonances observed in Figure 5a. It should be noted that conductive loss is only observable in the frequency range of 2.00–2.56 GHz. Thus, polarization relaxation contributes more to the dielectric loss than conductive loss in the composite of paraffin and (Ni@NO-C)_n_/NO-C.

Displayed in Figure 5c is the frequency dependence of the complex permeability of the composite of paraffin and (Ni@NO-C)_n_/NO-C in the frequency range of 2–18 GHz. The real part of complex permeability depicts the magnetic energy storage, and the imaginary part of permeability stands for energy conversion. It is obvious that the real part (*μ*′) decreases in a fluctuating fashion from 1.12 at 2 GHz to 1.04 at 8.32 GHz, and then increases in an oscillating fashion to 1.23 at 18 GHz, whereas the imaginary part (*μ*″) increases in a fluctuating manner from 0.078 at 2 GHz to 0.21 at 4.48 GHz and then decreases in the same way to 0.072 at 18 GHz. Thus, both the real part (*μ*′) and imaginary part (*μ*″) demonstrate multiple resonance processes in the frequency range of 2–18 GHz. In addition, the fluctuation amplitude of the value of *μ*″ is much smaller than that of *μ*′ in the frequency range of 8–18 GHz, indicating that the as-prepared EM absorbent also has a relatively stable magnetic loss ability. 

According to the related theory of magnetic loss, magnetic loss involves a series of complex mechanisms, mainly including eddy current loss, exchange resonance, natural resonance, domain wall resonance, and hysteresis loss. Since the domain wall resonance and hysteresis losses are negligible in the gigahertz range, the permeability of the composite of paraffin and (Ni@NO-C)_n_/NO-C might be mainly owing to eddy current loss, natural resonance, and exchange resonance rather than magnetic hysteresis or domain wall resonance. In order to explore the magnetic loss type of the sample, the eddy current coefficient *C*_0_ is calculated by the following equation [41]:

(2) $$C_{0}=\mu^{\prime \prime }{\left(\mu^{\prime }\right)}^{-2}f^{-1}$$

where *f* is the frequency of incident electromagnetic waves. If the eddy current effect is the only origin of magnetic loss, the value of *C*_0_ will be a constant in the corresponding frequency range [42]. As shown in Figure 5d, the value of *C*_0_ fluctuates greatly in the frequency range of 2.0–18.0 GHz, indicating that natural resonance and exchange resonance contribute greatly to the magnetic loss mechanism. As a matter of fact, the nine peaks observed in Figure 5c are indicative of various ferromagnetic resonance modes. The relatively sharp resonance peaks between 2.0 and 11.0 GHz are attributed to the natural resonance from static magnetic energy, while the relatively broad resonance peaks between 11.0 and 18.0 GHz belong to exchange resonance among different magnetic nanoparticles [43]. Natural resonance occurs when the external magnetic field frequency is the same as the inherent frequency of the (Ni@NO-C)_n_/NO-C, which is affected by the inherent properties of the material itself, such as size, morphology, and other factors. The multiple natural resonances of (Ni@NO-C)_n_/NO-C can be attributed to the discrete distributions of different-sized NO-C-encapsulated Ni@NO-C nanoparticles and their (Ni@NO-C)_n_ chains. On the other hand, exchange resonance is usually associated with spin-wave excitations, small size effects, and the surface effects of small-sized magnetic nanoparticles in the higher frequency range [43]. For the as-prepared (Ni@NO-C)_n_/NO-C EM absorbent, the Ni cores (or short Ni chains) are insulated by nitrogen and oxygen co-doped graphite layers, which can intensely suppress the eddy current effect. Therefore, the magnetic losses of the as-prepared (Ni@NO-C)_n_/NO-C EM absorbent mainly originate from natural resonance and exchange resonance.

In contrast to the arc-discharged Ni@CNOs particles [44] reported previously, the (Ni@NO-C)_n_/NO-C EM absorbent demonstrates a positive value of imaginary part (*μ*″), indicative of its more effective magnetic dissipation capacity. Under the action of an alternating electromagnetic field, the magnetic domain of a zero-dimensional particle is stationary. When the nickel particles are sequentially connected to form a one-dimensional chain, the magnetic domains of the different-sized nickel chains evolve with the change in the frequency of magnetic field due to the anisotropy of the nickel chains, which contributes largely to the enhancement of the natural resonance of (Ni@NO-C)_n_/NO-C EM absorbent. Furthermore, there are stronger magnetic interactions among (Ni@NO-C)_n_ chains than among Ni@CNOs particles, which lead to a larger exchange resonance among (Ni@NO-C)_n_ chains. Therefore, the larger magnetic energy loss of the (Ni@NO-C)_n_/NO-C absorbers than those of arc-discharged Ni@CNOs ones could be attributed to the enhancement of both natural resonance and exchange resonance caused by (Ni@NO-C)_n_ chains and NO-containing functional groups.

**Figure 6:**
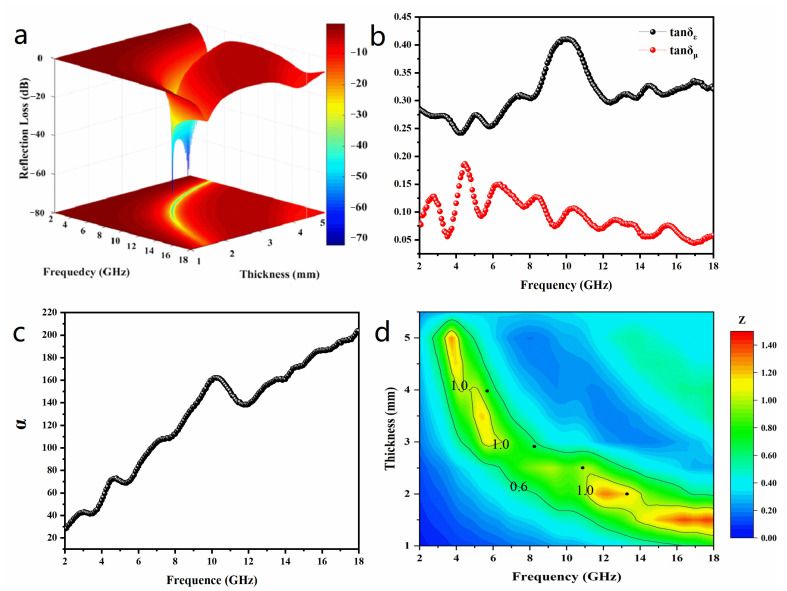
A 3D representation of the reflection loss values (**a**), dielectric loss tangent and magnetic loss tangent (**b**), attenuation constant *α* (**c**), and normalized impedance *Z* (**d**) of the 3D porous (Ni@NO-C)_n_/NO-C composite absorber.

The as-prepared (Ni@NO-C)_n_/NO-C absorbent exhibits a remarkable microwave absorption performance. Shown in Figure 6a is a 3D plot of reflection loss (RL) values versus frequency and thickness for the paraffin-based composite with 30 wt. % (Ni@NO-C)_n_/NO-C. The RL values are calculated based on transmit line theory according to the experimentally measured complex permittivity and permeability [10]. At a thickness of 2.0 mm, the effective absorption bandwidth reaches 4.3 GHz (11.7 to 16.0 GHz), and the maximum reflection loss reaches −20.3 dB at 13.28 GHz. When the thickness is 2.5 mm, the frequency band that can achieve effective absorption is from 8.4 to 12.4 GHz, and the maximum of reflection loss reaches −58.1 dB at 10.9 GHz. At a thickness of 2.9 mm, the effective absorption bandwidth reaches 4.2 GHz (6.9 to 11.1 GHz), and the maximum reflection loss reaches −72.3 dB at 8.2 GHz. When the thickness is 4.0 mm, the frequency band that can achieve effective absorption is from 5.4 to 7.4 GHz, and the maximum of reflection loss reaches −71.8 dB at 5.7 GHz. For comparison, Table 1 lists the microwave absorption data of typical Ni/carbon composite absorbers reported in recent years. It is obvious that the microwave absorption performance of the as-prepared (Ni@NO-C)_n_/NO-C absorber is superior to most of the reported Ni/carbon composite absorbers.

The excellent microwave absorption performance can be attributed to the cooperative consequences of dielectric loss and magnetic loss owing to the specific microstructure of the (Ni@NO-C)_n_/NO-C composite EM absorbent. To evaluate the loss ability of the electromagnetic energy, the dielectric loss tangent (tan *δ_ε_* = *ε*″/*ε*′) and magnetic loss tangent (tan *δ_μ_* = *μ*″/*μ*′) are calculated and shown in Figure 6b. It is obvious that tan *δ_ε_* is larger than tan *δ_μ_* in the measured frequency range, indicating that dielectric losses play more important roles than magnetic losses for the as-prepared (Ni@NO-C)_n_/NO-C absorbent. Moreover, multiple resonances are observed for tan *δ_ε_*, which do not completely appear in pairs with those of tan *δ_μ_* at the same frequency, implying an unbalanced energy conversion between complex permittivity and complex permeability [55]. In comparison with those magnetic metal/carbon composites reported previously [41,56,57,58], the as-prepared (Ni@NO-C)_n_/NO-C EM composite material exhibits the richest multiple resonances, which might be attributed to its special microstructure and the doping effect of nitrogen and oxygen in multi-layered graphite. From Figure 6b, it can also be seen that there are several maxima in the magnetic loss tangent curve, also indicating that the magnetic loss is mostly attributed to multiple magnetic resonances, i.e., natural resonance and exchange resonance. It is interesting to note that the as-prepared (Ni@NO-C)_n_/NO-C composite exhibits a much narrower gap between tan *δ_ε_* and tan *δ_μ_* than the arc-discharged Ni@CNOs composite nanoparticle [59] at the same frequency. Thus, it is expected that impedance matching would lead more radiated EM waves to enter the interiors of the as-prepared (Ni@NO-C)_n_/NO-C absorbent, which is beneficial for the subsequent absorption of EM waves.

A strong attenuation ability is a crucial factor for excellent EM wave absorption materials. The attenuation constant *α* determines the total loss ability of absorption materials and represents the amplitude attenuation of EM waves. The attenuation constant *α* of the as-prepared (Ni@NO-C)_n_/NO-C absorbent can be calculated by the following equation [60]:

(3) $$\alpha =\frac{\sqrt{2}\pi f}{c}\times \sqrt{(\mu^{\prime \prime }\varepsilon^{\prime \prime }-\mu^{\prime }\varepsilon^{\prime })+\sqrt{{\left(\mu^{\prime \prime }\varepsilon^{\prime \prime }-\mu^{\prime }\varepsilon^{\prime }\right)}^{2}+{\left(\mu \prime \varepsilon \prime \prime -\mu \prime \prime \varepsilon \prime \right)}^{2}}}$$

where *c* is the velocity of EM waves in free space. It can be seen from Figure 6c that the attenuation constant of the as-prepared (Ni@NO-C)_n_/NO-C absorbent increases from 28.0 at 2.0 GHz to 203.6 at 18.0 GHz with the increase in frequency, and the maxima appear at 4.8, 7.3, 10.2 and 13.5 GHz, etc. The attenuation constant value of the as-prepared (Ni@NO-C)_n_/NO-C absorbent is lower than that of the yolk−shell-structured Co_3_Fe_7_@C [17], which could be ascribed to its decrease in conductivity caused by nitrogen and oxygen doping. Nonetheless, it is still comparable to the waxberry-like hierarchical Ni@C microspheres [59].

Good impedance matching is another crucial factor for excellent EMW wave absorption materials. The coefficient of impedance matching determines whether the EM wave can enter the interior of the absorber, which is expressed by the following equation [61,62]:

(4) $$Z=\left|Z_{in}/Z_{0}\right|=\sqrt{\left|\mu_{r}/\varepsilon_{r}\right|}\tanh\left[j\left(2\pi fd/c\right)\sqrt{\mu_{r}/\varepsilon_{r}}\right]$$

where *Z_in_* is the input impedance, *Z*_0_ is the wave impedance in free space, *μ_r_* is the complex permeability, *ε_r_* is the complex permeability, *j* is the complex unit, *f* is the frequency, *d* is the thickness, and *c* is the velocity of EM waves in free space. When the input impedance *Z_in_* is numerically equal to the wave impedance in free space, the normalized input impedance *Z* value is equal to 1, and then the microwave can enter the absorbing material to the greatest extent. Therefore, the absorber shows better impedance matching when *Z* is close to 1. Displayed in Figure 6d are the 3D *Z* values of the as-prepared (Ni@NO-C)_n_/NO-C absorbent calculated in the thickness range of 1.0–5.5 mm and in the frequency range of 2–18 GHz. It is obvious that good impedance matching could be achieved for the as-prepared absorbent by adjusting thickness and frequency to get a *Z* value close to 1; the thinner the sample, the higher the impedance matching frequency. The four black dots observed in Figure 6d mark the values of thickness and frequency corresponding to the maxima of reflection loss and effective absorption bandwidth for the as-prepared samples (see Figure 6a), whose impedance matching coefficients (*Z*) are in the range of 0.60–1.18, showing the balance between attenuation capability and impedance matching.

## 4. Conclusions

In summary, a 3D porous (Ni@NO-C)_n_/NO-C composite absorber was prepared by the pyrolysis of Ni chains and melamine, whose unique microstructure contributes greatly to its quite good microwave wave absorption performance. The NO-C materials contribute a tailored dielectric loss through conductivity modification as well as multiple interfacial and intrinsic electric dipole polarizations, while the (Ni@NO-C)_n_ chains dispersed in the 3D porous networks of NO-C materials offer a proper magnetic loss owing to their natural and exchange resonances. Moreover, the multiple resonances observed in the curves of the dielectric loss tangent and magnetic loss tangent make it easy to achieve good impedance matching, which is beneficial for the improvement of the maximum reflection loss and the effective absorption bandwidth of the as-prepared 3D porous (Ni@NO-C)_n_/NO-C composite absorber. It is believed that the microwave absorption performances of the as-prepared absorber could be further improved by the complete optimization of its composition and microstructure, and the 3D porous (Ni@NO-C)_n_/NO-C composite material has the potential to be a lightweight microwave adsorption absorber with high efficiency and high stability. 

## Figures and Tables

**Table 1:** Comparison of the microwave absorptions of typical Ni/carbon composite absorbers.

Materials	Filler Load (wt. %)	Matching Thickness (mm)	RLmax (dB)	EAB (GHz)	Refs.
Ni/C hollow microspheres	30	1.80	−57.25	5.10	[45]
Ni@C nanorods	30	1.66	−58.7	4.4	[46]
Ni@CN nanocapsules	-	2.3	−35.8	≈3.5	[47]
NiCo@g-C_3_N_4_	20	2.0	−35.05	4.80	[48]
Ni/C	30	1.5	≈−17.6	4.8	[49]
Ni/C microsphere	75	1.8	−28.40	4.90	[50]
Ni@C nanorods	40	1.7	≈−22	5.2	[51]
Ni/C composite (s500)	40	2.60	−51.80	3.48	[52]
MXene/Ni/N-CNT (HM1)	-	1.49	−57.78	2.08	[53]
MXene@Ni-CZIF	50	3.4	−64.11	≈1.7	[54]
MXene@Ni-CZIF	33	4.8	−34.52	1.48	[54]
(Ni@NO-C)_n_/NO-C	30	2.5	−57.9	4.0	Herein
(Ni@NO-C)_n_/NO-C	30	2.9	−72.3	4.2	Herein

## Data Availability

The data presented in this study are available on request from the corresponding author.
